# Identification of potential candidate genes for hypertensive nephropathy based on gene expression profile

**DOI:** 10.1186/s12882-016-0366-8

**Published:** 2016-10-18

**Authors:** Zhi Chen, Hao Wu, Guohua Wang, Ye Feng

**Affiliations:** 1 Department of Nephrology, First Hospital of Jilin University, Jilin, 130021 China; 2 Department of Neonatology, First Hospital of Jilin University, Jilin, 130021 China; 3 Department of Gastrointestinal Colorectal and Anal Surgery, China-Japan Union Hospital of Jilin University, No.126 Xiantai Avenue, Jilin, 130033 China

**Keywords:** Hypertensive nephropathy, Differentially expressed gene, Pathway, Network, microRNA

## Abstract

**Background:**

This study was aimed to explore the molecular mechanisms of hypertensive nephropathy (HTN).

**Methods:**

Gene expression profile of GSE37460, which based on 27 healthy living donor samples (HTN group) and 15 hypertensive nephropathy samples (control group), were downloaded from Gene Expression Omnibus (GEO) database. The differentially expressed genes (DEGs) between two groups were identified. STRING database was used to reveal protein-protein interaction (PPI) network of DEGs, followed by the functional enrichment analysis of the PPI network. Additionally, miRNA-DEG regulatory network was constructed to reveal the validated miRNAs targeting the DEGs.

**Results:**

In total, 51 up-regulated genes and 140 down-regulated genes were obtained. In the PPI network, cytochrome P450 3A4 (*CYP3A4*) and angiotensin II receptor type 1 *(AGTR1*) had a higher degree, and *CYP3A4* interacted with *CYP4A11*. The DEGs in the network were significantly enriched in drug metabolism, focal adhesion and arachidonic acid metabolism. Furthermore, in the miRNA-DEG regulatory network, hsa-miR-335-5p and hsa-miR-26b-5p were the two most outstanding miRNAs. *AGTR1*, *CYP3A4* and *CYP4A11* were predicted to be regulated by hsa-miR-26b-5p.

**Conclusion:**

The DEGs, such as *AGTR1*, *CYP3A4* and *CYP4A11* may play critical roles in the development of HTN likely via the regulation by hsa-miR-26b-5p and taking part in some pathways.

## Background

Hypertensive nephropathy (HTN) is a kind of the kidney injury due to chronic high blood pressure [1]. Hypertension-induced renal damage is an increasingly common disease recently, and approximately 25 % of patients currently treated with dialysis are hypertensive before renal replacement therapy started [2]. Although the antihypertensive drugs like cilnidipine (2-methoxyethyl cinnamyl 2,6-dimethyl-4-(3-nitrophenyl)-1,4-dihydropyridine-3,5-dicarboxylate) and avosentan (N-[6-methoxy-5-(2-methoxyphenoxy)-2-(pyridin-4-yl)pyrimidin-4-yl]-5-methylpyridine-2-sulfonamide) are commonly used for the treatment of HTN [3, 4], the effect of clinical treatment for HTN is still not ideal [5]. Due to the increasing morbidity and mortality of renal disease, molecular mechanisms of HTN are urgently required to be revealed, which contributes to the improvement of therapeutic strategies to control blood pressure and delay progression of HTN [6].

Recently, the studies based on gene or protein investigation are successfully used to reveal the potential mechanisms of HTN. For instance, using distinct lines of the spontaneously hypertensive rat, Dmitrieva et al*.* have shown a major change in transcriptional control by hepatocyte nuclear factor 1 that affects pathways like redox and other genes, which further lead to the hypertensive renal injury [7]. Periostin, also called osteoblast-specific factor 2, strongly associated with plasma creatinine, proteinuria and renal blood flow, has been identified as a critical marker of progression and regression in HTN [8]. Moreover, SMAD family member 7 has also been discovered to inhibit AngII-mediated HTN through the Sp1/SMAD family member 3/nuclear factor kappa B (NF-κB)/miR-29b regulatory network, and it is identified as a therapeutic biomarker for AngII-mediated HTN [9]. Furthermore, the role of microRNAs (miRNAs) in HTN has also been investigated in recent years. A set of miRNAs (e.g. miR-429, miR-200a, miR-205, miR-200b, miR-141, and miR-192) have been found to be highly expressed in hypertensive nephrosclerosis, and the degree of upregulation is closely related to disease severity [10]. Hsa-miR-181a has confirmed to regulate *REN* (renin) and apoptosis-inducing factor, mitochondrion-associated, 1 mRNA, and modulate *REN* expression in HTN [11]. Using a mRNA expression profiling dataset GSE37460, Berthier et al*.* have discovered a series of pathways, such as endothelial cell activation/injury, immune cell infiltration/activation, and tissue remodeling/fibrosis, with macrophage/dendritic cell activation in both murine models and human lupus nephritis, and they have also found that nuclear factor κB1 and peroxisome proliferator-activated receptor γare major regulatory nodes in the tubulointerstitial and glomerular networks [12]. However, the differences between human HTN and healthy controls remain unclear, and more genes and pathways associated with HTN have not been found.

In the present study, based on the expression profile data of healthy living donor samples and HTN samples deposited by Berthier et al*.* [12], a bioinformatics analysis was performed. After identification of differentially expressed genes (DEGs) and functional enrichment analysis of the DEGs, protein-protein interactions (PPIs) of the DEGs were analyzed. Furthermore, miRNAs that regulate DEGs were further investigated. These results may contribute to a better understanding of the molecular mechanisms of HTN pathogenesis, and provide valid biological information for further investigation of HTN.

## Methods

As the study did not involve any human or animals, the ethical approval was not required.

### Affymetrix microarray data

The mRNA expression profile of GSE37460 [12] was downloaded from a public functional genomics data repository GEO (Gene Expression Omnibus, http://www.ncbi.nlm.nih.gov/geo/), which was based on two platforms, including Affymetrix Human Genome U133 Plus 2.0 Array (GPL11670) and Affymetrix GeneChip Human Genome HG-U133A Custom Array (GPL14663) (Affymetrix, California, USA). Glomeruli from kidney biopsy samples from 27 healthy living donors (control group), 15 hypertensive nephropathy (HTN group) and 25 IgA nephropathy (IgAN group) participants were included in this profile. To explore the abnormal transcription of HTN, the samples from HTN group and healthy control group were specially enrolled for the following investigation.

The data in the CEL source files were normalized by using the R package CONOR [13], including background correction, quantile normalization and probe summarization.

### Identification of DEGs

The DEGs between control group and HTN group were analyzed by using the Linear Models for Microarray Data (LIMMA, http://www.bioconductor.org/packages/release/bioc/html/limma.html) package in Bioconductor software [14]. The raw p-value of each gene was adjusted into the false discovery rate (FDR) by using Bonferroni correction [15]. Only the genes with FDR-value < 0.05 and |log_2_FC (fold change)| ≥ 1.5 were identified as DEGs.

### Construction of PPI network

The Search Tool for the Retrieval of Interacting Genes/Proteins (STRING) database includes known and predicted PPIs [16]. The interactions of proteins encoded by DEGs were selected as the background network according to STRING v9.1 database with combined score > 0.9. Degree (the number of interactions linked to one target) was used to describe the frequency of interactions. Subsequently, the DEGs and related first neighbors were further extracted to construct the PPI network, which was visualized by Cytoscape (http://www.cytoscape.org/) [17].

### Functional enrichment analysis of pathways for genes in the PPI network

The plugin in Cytoscape software, ClueGO, can integrate Gene Ontology (GO) terms and Kyoto Encyclopedia of Genes and Genomes (KEGG) pathways to create a functionally organized network [18]. By calculating the kappa coefficient [19], the functional pathways can be divided into several function groups in the network. To reveal the biological functions of genes in the PPI network, the KEGG pathway enrichment analysis for DEGs in the PPI network was performed based on ClueGO. The raw *p*-value of each pathway term was adjusted into the FDR by using Bonferroni correction [15]. FDR-value < 0.05 was considered as the cutoff criterion of significant difference, and pathway groups were generated based on kappa = 0.4.

### Analysis of miRNA regulation factor

The multiMiR (http://multimir.ucdenver.edu/) is an integration of miRNA-target interactions in R package and database [20]. MultiMiR includes 3 validated miRNA-target databases (miRecords, miRTarBase and TarBase), 8 predicted miRNA-target databases (miRDB, PicTar, DIANA-microT, ElMMo, PITA and TargetScan) and 3 disease- and drug-related miRNA databases (miR2Disease, PharmacomiR and PhenomiR). In the present study, the validated miRNAs of DEGs were investigated based on multiMiR.

## Results

### Identification of DEGs

**Fig. 1:**
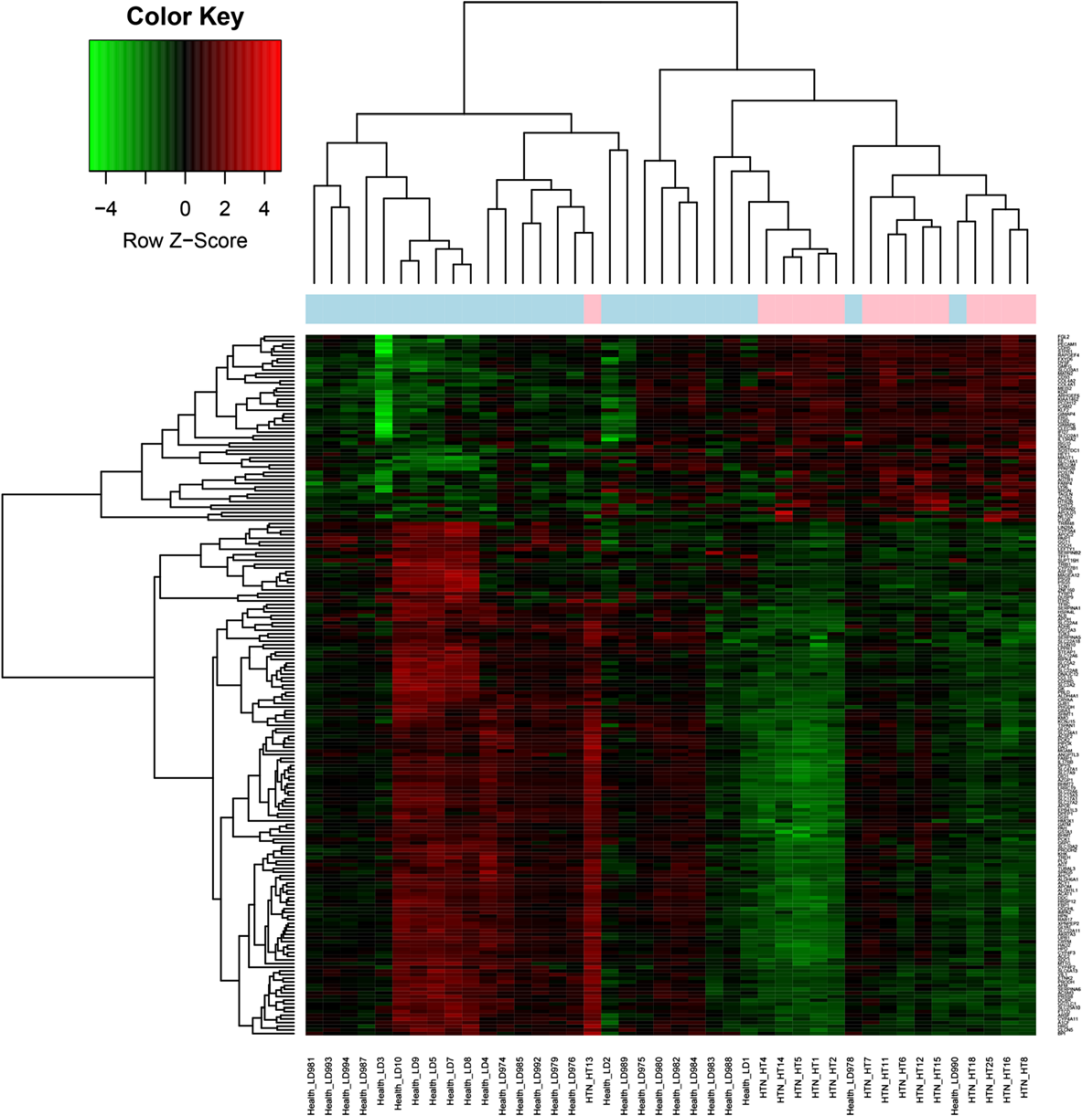
Heat map for the differentially expressed genes. Each row represents a single gene; each column represents a tissue sample. Green blocks represent the downregulated differentially expressed genes; red blocks represent the upregulated differentially expressed genes; black blocks represent non-significant genes; light blue represents the control group, while pink represents hypertensive nephropathy group

A large number of calculations were performed, and the original data were analyzed and filtered. A total of 51 up-regulated DEGs and 140 down-regulated DEGs were obtained with thresholds of FDR < 0.05 and |log_2_FC| ≥ 1.5. The heat map of DEGs showed that the HTN samples were distinguished clearly from the healthy samples by the identified DEGs (Fig. 1).

### PPI network investigation and functional enrichment analysis

**Fig. 2:**
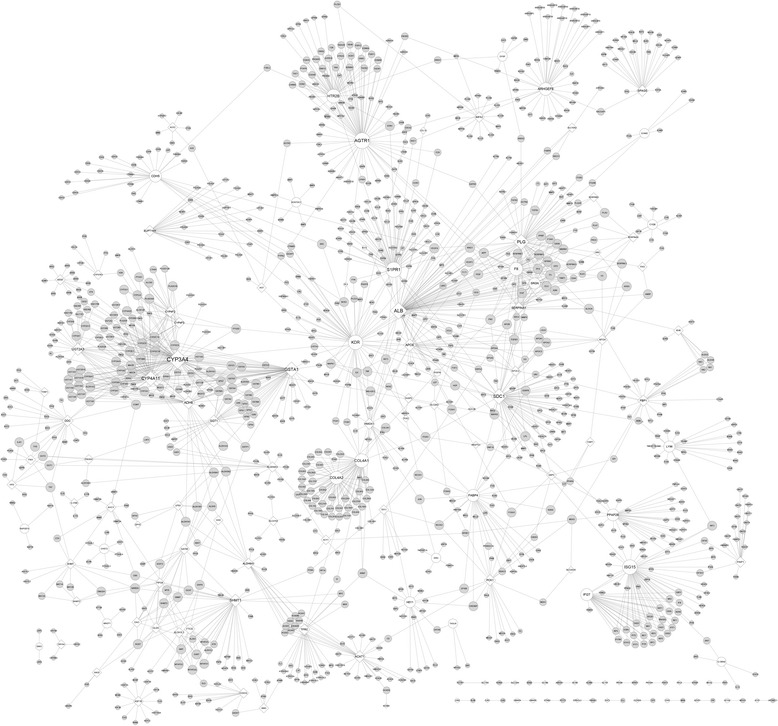
Protein-protein interaction network consisting of differentially expressed genes and non-significant genes. White diamonds represent the down-regulated genes; white round nodes represent the up-regulated genes; gray round nodes represent the non-significant genes. The node size is proportional to the degree value

With the combined score > 0.9, a total of 1220 nodes (36 up-regulated, 88 down-regulated and 1096 non-significant ones) were included in the PPI network (Fig. 2). The average degree for all enrolled DEGs was 14.5. The genes with nodes degree value > 50 [e.g. cytochrome P450 family 3 subfamily A member 4 (*CYP3A4*) and angiotensin II receptor type 1 (*AGTR1*)] were listed in Table 1.

**Table 1:** The up- and down-regulated differentially expressed genes in the protein-protein interaction network (degree > 50 are listed)

Gene symbol	State	Degree value
*CYP3A4*	Down-regulated	89
*ALB*	Down-regulated	71
*AGTR1*	Up-regulated	64
*SDC1*	Down-regulated	59
*PLG*	Down-regulated	57
*S1PR1*	Up-regulated	57
*GSTA1*	Down-regulated	56
*CYP4A11*	Down-regulated	55
*ISG15*	Up-regulated	52
*KDR*	Up-regulated	50

**Fig. 3:**
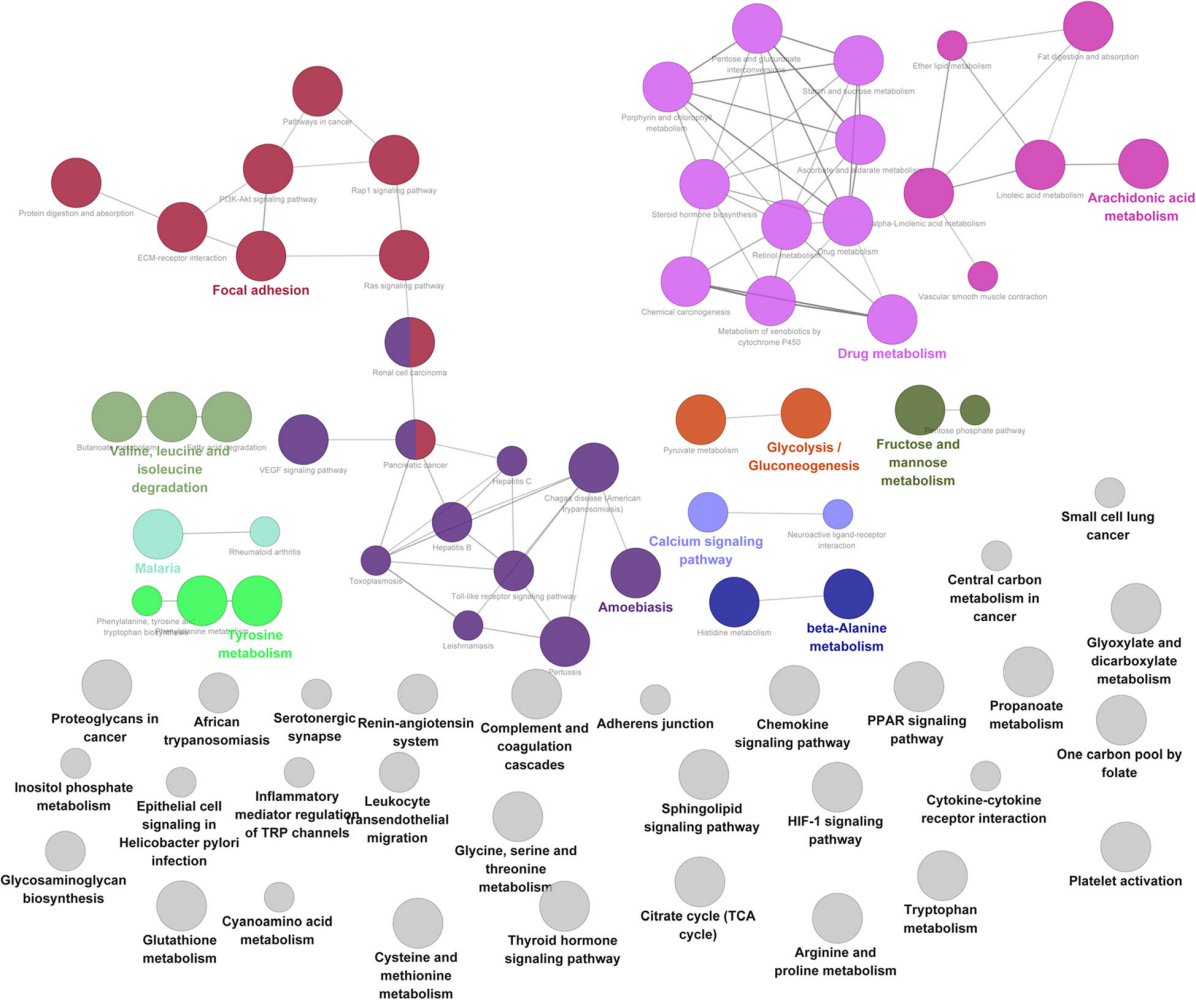
KEGG pathway enrichment analysis for differentially expressed genes in protein-protein interaction network. Each node is a KEGG pathway item, and node size is proportional to the pathway significance. Edge between nodes mean that they share common genes, and the width of the edge is proportional to the number of common genes. Pathways are classified into several functional groups (different node color) accordance with the kappa value. The most significant pathway of each group labels with a highlighted color. KEGG, Kyoto Encyclopedia Of Genes And Genomes

Furthermore, with FDR < 0.05 and kappa = 0.4, the KEGG pathways enriched by DEGs in the present PPI network were performed based on ClueGO (Fig. 3). The DEGs were significantly enriched in pathways, such as drug metabolism [e.g. *CYP3A4* and alcohol dehydrogenase 1A (class I), alpha polypeptide (*ADH1A*)], focal adhesion [e.g. collagen type IV alpha 1 (*COL4A1*)], and arachidonic acid metabolism [e.g. cytochrome P450 family 2 subfamily B member 6 (*CYP2B6*) and cytochrome P450 family 4 subfamily A member 11 (*CYP4A11*)] (Table 2).

**Table 2:** The result of the most significant KEGG pathway in each functional group

ID	Pathway name	Count	*p*-value	FDR	Genes
00982	Drug metabolism	60	8.02E-43	1.93E-40	*CYP3A4*, *ADH1A*, *FMO1*, *GSTA1*, *MAOA* …
00350	Tyrosine metabolism	29	1.36E-19	3.19E-17	*ADH1A*, *COMT*, *DBH*, *FAH*, *GOT1*…
04510	Focal adhesion	79	3.98E-18	9.23E-16	*ACTN1*, *ACTN2*, *COL4A1*, *EGF*, *VWF*…
00010	Glycolysis/Gluconeogenesis	38	4.46E-16	1.02E-13	*ACSS1*, *DLAT*, *ENO2*, *FBP1*, *HK1*…
05146	Amoebiasis	49	6.08E-15	1.38E-12	*ACTN1*, *CD14*, *FN1*, *IL10*, *TNF*…
00590	Arachidonic acid metabolism	35	8.03E-15	1.81E-12	*ALOX12B*, *CYP2B6*, *CYP2B6*, *GGT1*, *CYP4A11*…
00280	Valine, leucine and isoleucine degradation	29	6.82E-14	1.53E-11	*AACS*, *DLD*, *EHHADH*, *IL4I1*, *PCCB*…
00410	beta-Alanine metabolism	21	1.48E-11	3.22E-09	*ABAT*, *ACADM*, *DPYD*, *EHHADH*, *UPB1*…
00051	Fructose and mannose metabolism	20	8.95E-10	1.87E-07	*AKR1B1*, *FBP1*, *HK1*, *MPI*, *PFKFB1*…
05144	Malaria	22	2.04E-07	3.90E-05	*CCL2*, *HGF*, *ICAM1*, *MET*, *VCAM1*…
04020	Calcium signaling pathway	47	1.59E-05	0.002881	*ADCY2*, *BDKRB1*, *AGTR1*, *EDNRA*, *F2R*…

*KEGG* Kyoto Encyclopedia of Genes and Genomes, *FDR* false discovery rate. The *p*-value is adjusted into FDR using the Bonferroni correction

### The miRNA-DEG regulatory network investigation

**Fig. 4:**
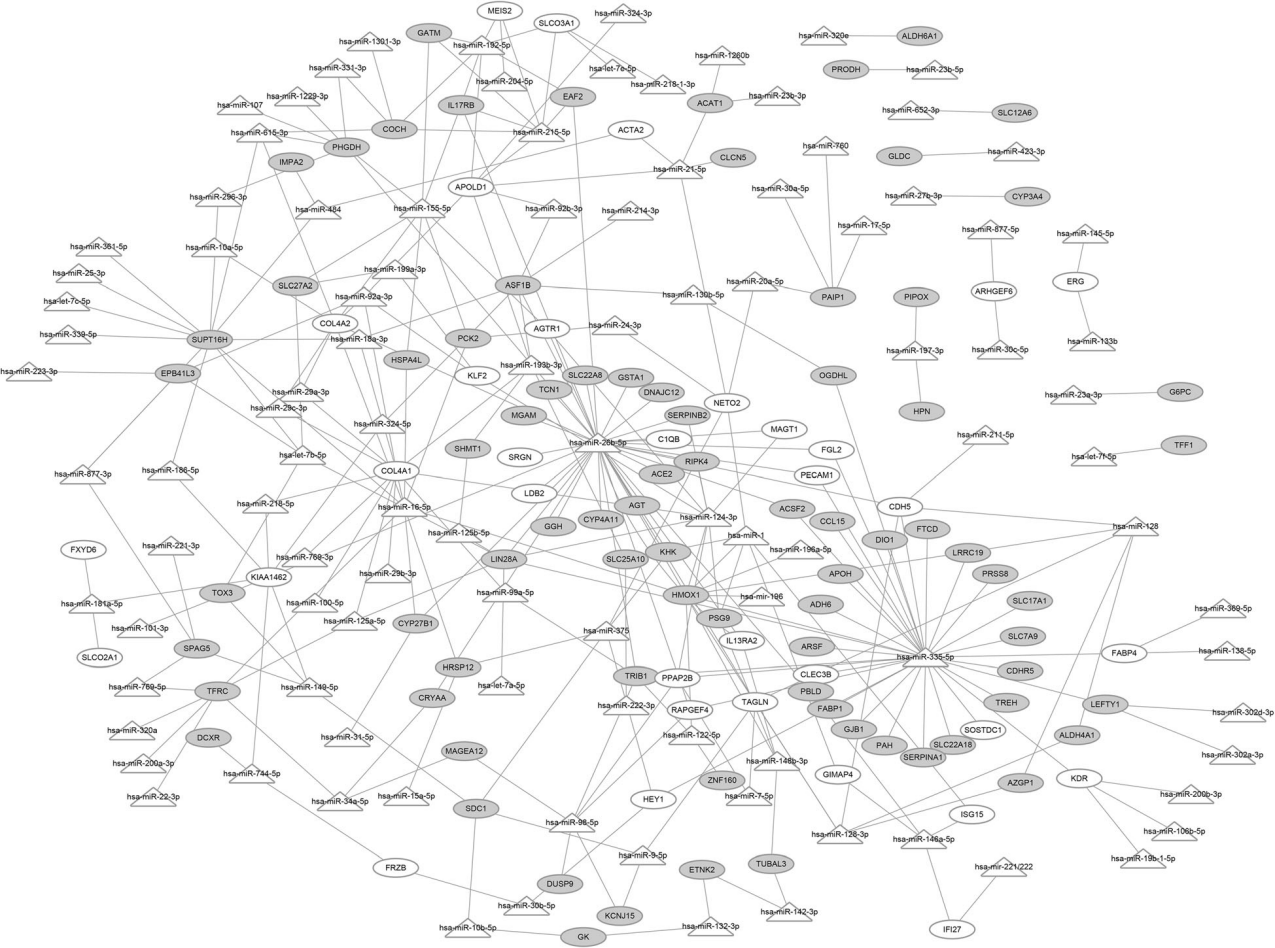
The microRNA-differentially expressed gene regulatory network. White triangles represent microRNAs; white ellipses represent the up-regulated genes; grey ellipses represent the down-regulated genes

To study the validated miRNAs of DEGs, the miRNA-DEG regulatory network was constructed based on multiMiR software. A total of 217 nodes (103 miRNAs, 34 up-regulated DEGs and 80 down-regulated DEGs) and 295 interactions were included in this network (Fig. 4). Among the miRNAs, hsa-miR-335-5p and hsa-miR-26b-5p modulated the majority of DEGs in this network. For example, hsa-miR-26b-5p regulated *COL4A1*, *CYP4A11* and *AGTR1*.

## Discussion

HTN is an increasingly common kidney disease in patients with hypertension recently [6]. However, the potential mechanisms of the progress of HTN is still unclear. In this study, a bioinformatics analysis of gene expression profile for healthy living donor samples and HTN samples was performed to explore the mechanisms of HTN. In total, 51 up-regulated DEGs and 140 down-regulated DEGs were identified in the HTN samples compared with the healthy controls. The DEGs were significantly enriched in pathways like drug metabolism, focal adhesion and arachidonic acid metabolism. Furthermore, in the miRNA-DEG regulatory network, hsa-miR-335-5p and hsa-miR-26b-5p were the two most outstanding miRNAs.

In the present study, *CYP3A4* and *AGTR1* were the outstanding down- and up-regulated DEGs with the highest degree in the PPI network, respectively. *CYP3A4* encodes an enzyme belonging to the cytochrome P450 (CYP P450) superfamily, which is a group of heme-thiolate monooxygenases and participate in a variety of oxidation reactions [21]. CYP P450 expression can be altered by inflammation [22], which is involved in renal injury [23]. The expression of *CYP3A4* is induced by glucocorticoids and involved in the metabolism of multiple drugs [24]. A previous study has reported that *CYP3A4* T16090C SNP responses to amlodipine among African-Americans with early HTN [25]. Moreover, the expression of *CYP3A4* is elevated in patients with end-stage renal disease [26]. *CYP4A11*, a homologue of *CYP3A4*, had a higher degree in the PPI network and interacted with *CYP4A11*. In this study, *CYP4A11* was significantly enriched in the pathway of arachidonic acid metabolism. CYP P450 metabolites of arachidonic acid play an important role in the control of blood pressure, chronic kidney disease through the maintenance of the glomerular permeability barrier to albumin [27, 28]. Furthermore, 20-hydroxyeicosatetraenoic acid (20-HETE) has renoprotective actions in hypertension, and mutations in *CYP4A11* that produces 20-HETE have been linked to elevated blood pressure in humans [29, 30], indicating the important role of *CYP4A11* in HTN. In the present study, *CYP4A11* was predicted to be regulated by hsa-miR-26b-5p. An recent study has demonstrated that expression of miR-26b-5p is significantly decreased in basal serum samples from those patients with acute kidney injury, and it is a diagnostic biomarker of acute kidney injury [31]. Currently, there is no any other evidence to prove the associations of *CYP3A4*, *CYP4A11* and hsa-miR-26b-5p with HTN. Given the above studies, we speculated that *CYP3A4* and *CYP4A11*, as well as hsa-miR-26b-5p may play pivotal roles in the progress of HTN.

*AGTR1* also had a higher degree in the PPI network, and it was modulated by hsa-miR-26b-5p. *AGTR1* encodes of angiotensin II type 1 receptor, which is an important effector in the control of blood pressure [32]. Previous studies have shown that variants on genes including *AGTR1* are associated with hypertension [33, 34]. The (−535) T allele of *AGTR1* is believed to increase hypertension risk among African Americans [35]. Moreover, *AGTR1* polymorphisms are believed to be associated with the renal function [36, 37]. Durvasula et al*.* have reported that intrarenal production of angiotensin II plays an important role in mediating HTN through inducing podocyte injury and promoting the development of glomerulosclerosis [38]. Furthermore, a previous study has found that angiotensin II-induced arterial hypertension and vascular dysfunction are mediated by lysozyme M–positive monocytes [39], which participate in renal injury [40]. Although there is no direct evidence to prove the association of *AGTR1* and HTN, we speculate that *AGTR1* may exert critical functions in the progress of HTN.

However, this study has several limitations. The major limitation is that the aforementioned results should be validated by other microarray data or experimental studies, which will be conducted and reported later. Furthermore, more patients with HTN should be included for the analysis. Additionally, the clinical data of the patients are not available, thus the patients may be heterogenous. In the further study, more samples from patients with HTN will be used for the verification experiments to confirm our results.

## Conclusion

In conclusion, 51 up-regulated DEGs and 140 down-regulated DEGs were identified in the HTN samples compared with the healthy controls. The DEGs such as *CYP3A4*, *CYP4A11* and *AGTR1*, may be crucial in the progress of HTN, via the regulation by miRNAs (e.g. hsa-miR-26b-5p) and participation in the biological pathways (e.g. arachidonic acid metabolism). Notably, the above discussed genes and miRNA are new-found to be correlated with HTN in this study, and they are worth further investigation. These findings provide new information for further experimental studies. If these genes and miRNAs are confirmed by experiments, they will be promising to be used in the diagnosis or clinical therapy of HTN.

## References

[1] Toto RB (2003). Nephrology Forum - Hypertensive nephrosclerosis in African Americans. Kidney Int.

[2] Rutkowski B, Tylicki L, Manitius J, Lysiak-Szydlowska W (1999). Hypertensive nephropathy - an increasing clinical problem. Miner Electrolyte Metab.

[3] Uchida S, Takahashi M, Sugawara M, Saito T, Nakai K, Fujita M (2014). Effects of the N/L-type calcium channel blocker cilnidipine on nephropathy and uric acid metabolism in hypertensive patients with chronic kidney disease (J-CIRCLE study). J Clin Hypertens.

[4] Baltatu OC, Zaugg CE, Schumacher C, Louie P, Campos LA, Bader M (2014). Avosentan is protective in hypertensive nephropathy at doses not causing fluid retention. Pharmacol Res.

[5] Wang XC, Liu CH, Chen YJ, Wu Y, Yang LS, Liu HM (2013). Clinical and pathological analysis of the kidney in patients with hypertensive nephropathy. Exp Ther Med.

[6] Hart PD, Bakris GL (2010). Hypertensive nephropathy: prevention and treatment recommendations. Expert Opin Pharmacother.

[7] Dmitrieva RI, Hinojos CA, Boerwinkle E, Braun MC, Fornage M, Doris PA (2008). Hepatocyte nuclear factor 1 and hypertensive nephropathy. Hypertension.

[8] Guerrot D, Dussaule J-C, Mael-Ainin M, Xu-Dubois Y-C, Rondeau E, Chatziantoniou C (2012). Identification of periostin as a critical marker of progression/reversal of hypertensive nephropathy. PLoS One.

[9] Liu G-X, Li Y-Q, Huang XR, Wei LH, Zhang Y, Feng M (2014). Smad7 inhibits AngII-mediated hypertensive nephropathy in a mouse model of hypertension. Clin Sci.

[10] Wang G, Kwan BC-H, Lai FM-M, Choi PC-L, Chow K-M, Li PK-T (2010). Intrarenal expression of miRNAs in patients with hypertensive nephrosclerosis. Am J Hypertens.

[11] Marques FZ, Campain AE, Tomaszewski M, Zukowska-Szczechowska E, Yang YHJ, Charchar FJ (2011). Gene expression profiling reveals renin mRNA overexpression in human hypertensive kidneys and a role for microRNAs. Hypertension.

[12] Berthier CC, Bethunaickan R, Gonzalez-Rivera T, Nair V, Ramanujam M, Zhang W (2012). Cross-species transcriptional network analysis defines shared inflammatory responses in murine and human lupus nephritis. J Immunol.

[13] Warnat P, Eils R, Brors B (2005). Cross-platform analysis of cancer microarray data improves gene expression based classification of phenotypes. BMC bioinformatics.

[14] Ritchie ME, Phipson B, Wu D, Hu Y, Law CW, Shi W (2015). limma powers differential expression analyses for RNA-sequencing and microarray studies. Nucleic Acids Res.

[15] Armstrong RA (2014). When to use the Bonferroni correction. Ophthalmic Physiol Opt.

[16] Franceschini A, Szklarczyk D, Frankild S, Kuhn M, Simonovic M, Roth A (2013). STRING v9.1: protein-protein interaction networks, with increased coverage and integration. Nucleic Acids Res.

[17] Shannon P, Markiel A, Ozier O, Baliga NS, Wang JT, Ramage D (2003). Cytoscape: a software environment for integrated models of biomolecular interaction networks. Genome Res.

[18] Bindea G, Mlecnik B, Hackl H, Charoentong P, Tosolini M, Kirilovsky A (2009). ClueGO: a Cytoscape plug-in to decipher functionally grouped gene ontology and pathway annotation networks. Bioinformatics.

[19] Ramos H, Shannon P, Aebersold R (2008). The protein information and property explorer: an easy-to-use, rich-client web application for the management and functional analysis of proteomic data. Bioinformatics.

[20] Ru Y, Kechris KJ, Tabakoff B, Hoffman P, Radcliffe RA, Bowler R (2014). The multiMiR R package and database: integration of microRNA-target interactions along with their disease and drug associations. Nucleic Acids Res.

[21] Werck-Reichhart D, Feyereisen R (2000). Cytochromes P450: a success story. Genome Biol.

[22] Theken KN, Deng Y, Kannon MA, Miller TM, Poloyac SM, Lee CR (2011). Activation of the acute inflammatory response alters cytochrome P450 expression and eicosanoid metabolism. Drug Metab. Dispos..

[23] Daemen MA, De VB, Buurman WA (2002). Apoptosis and inflammation in renal reperfusion injury. Transplantation.

[24] Zhang H, Coville PF, Walker RJ, Miners JO, Birkett DJ, Wanwimolruk S (1997). Evidence for involvement of human CYP3A in the 3-hydroxylation of quinine. Br J Clin Pharmacol.

[25] Bhatnagar V, Garcia EP, O’Connor DT, Brophy VH, Alcaraz J, Richard E (2010). CYP3A4 and CYP3A5 polymorphisms and blood pressure response to amlodipine among African-American men and women with early hypertensive renal disease. Am J Nephrol.

[26] Tsujimoto M, Nagano Y, Hosoda S, Shiraishi A, Miyoshi A, Hiraoka S (2013). Effects of decreased vitamin D and accumulated uremic toxin on human CYP3A4 activity in patients with end-stage renal disease. Toxins.

[27] Williams JM, Sharma M, Anjaiahh S, Falck JR, Roman RJ (2007). Role of endogenous CYP450 metabolites of arachidonic acid in maintaining the glomerular protein permeability barrier. Am. J. Physiol. Ren. Physiol..

[28] Fan F, Muroya Y, Roman RJ (2015). Cytochrome P450 eicosanoids in hypertension and renal disease. Curr Opin Nephrol Hypertens.

[29] Liang J, Yan M, Yang L, Suyila Q, Cui H, Su X (2014). Association of a CYP4A11 polymorphism and hypertension in the Mongolian and Han populations of China. Genet Mol Res.

[30] Elijovich F, Laffer CL (2008). The relationship between CYP4A11 and human hypertension. J Hypertens.

[31] Aguado-Fraile E, Ramos E, Conde E, Rodríguez M, Martín-Gómez L, Lietor A (2015). A Pilot Study Identifying a Set of microRNAs As Precise Diagnostic Biomarkers of Acute Kidney Injury. PLoS One.

[32] Su X, Lee L, Li X, Lv J, Hu Y, Zhan S (2007). Association between angiotensinogen, angiotensin II receptor genes, and blood pressure response to an angiotensin-converting enzyme inhibitor. Circulation.

[33] Valencia DM, Naranjo CA, Parra MV, Caro MA, Valencia AV, Jaramillo CJ (2013). Association and interaction of AGT, AGTR1, ACE, ADRB2, DRD1, ADD1, ADD2, ATP2B1, TBXA2R and PTGS2 genes on the risk of hypertension in Antioquian population. Biomedica.

[34] Sun Y, Liao Y, Yuan Y, Feng L, Ma S, Wei F (2014). Influence of autoantibodies against AT1 receptor and AGTR1 polymorphisms on candesartan-based antihypertensive regimen: results from the study of optimal treatment in hypertensive patients with anti-AT1-receptor autoantibodies trial. J Am Soc Hypertens.

[35] Henderson SO, Haiman CA, Mack W (2004). Multiple Polymorphisms in the renin- angiotensin-aldosterone system (ACE, CYP11B2, AGTR1) and their contribution to hypertension in African Americans and Latinos in the multiethnic cohort. Am J Med Sci.

[36] Smilde TD, Zuurman MW, Hillege HL, van Veldhuisen DJ, van Gilst WH, van der Steege G (2007). Renal function dependent association of AGTR1 polymorphism (A1166C) and electrocardiographic left-ventricular hypertrophy. Am J Hypertens.

[37] Campbell CY, Fang BF, Guo X, Peralta CA, Psaty BM, Rich SS (2010). Associations between genetic variants in the ACE, AGT, AGTR1 and AGTR2 genes and renal function in the Multi-ethnic Study of Atherosclerosis. Am J Nephrol.

[38] Durvasula RV, Shankland SJ (2006). The renin-angiotensin system in glomerular podocytes: mediator of glomerulosclerosis and link to hypertensive nephropathy. Curr Hypertens Rep.

[39] Wenzel P, Knorr M, Kossmann S, Stratmann J, Hausding M, Schuhmacher S (2011). Lysozyme M-positive monocytes mediate angiotensin II-induced arterial hypertension and vascular dysfunction. Circulation.

[40] Li L, Huang L, Sung SSJ, Vergis AL, Rosin DL, Jr CER (2008). The chemokine receptors CCR2 and CX3CR1 mediate monocyte/macrophage trafficking in kidney ischemia–reperfusion injury. Kidney Int.

